# Supplementary figures and images for: A combined EM and proteomic analysis places HIV-1 Vpu at the crossroads of retromer and ESCRT complexes: PTPN23 is a Vpu-cofactor
Source: PLoS Pathog. 2021 Nov 29;17(11):e1009409. doi: 10.1371/journal.ppat.1009409 (PMC8659692; doi:10.1371/journal.ppat.1009409)

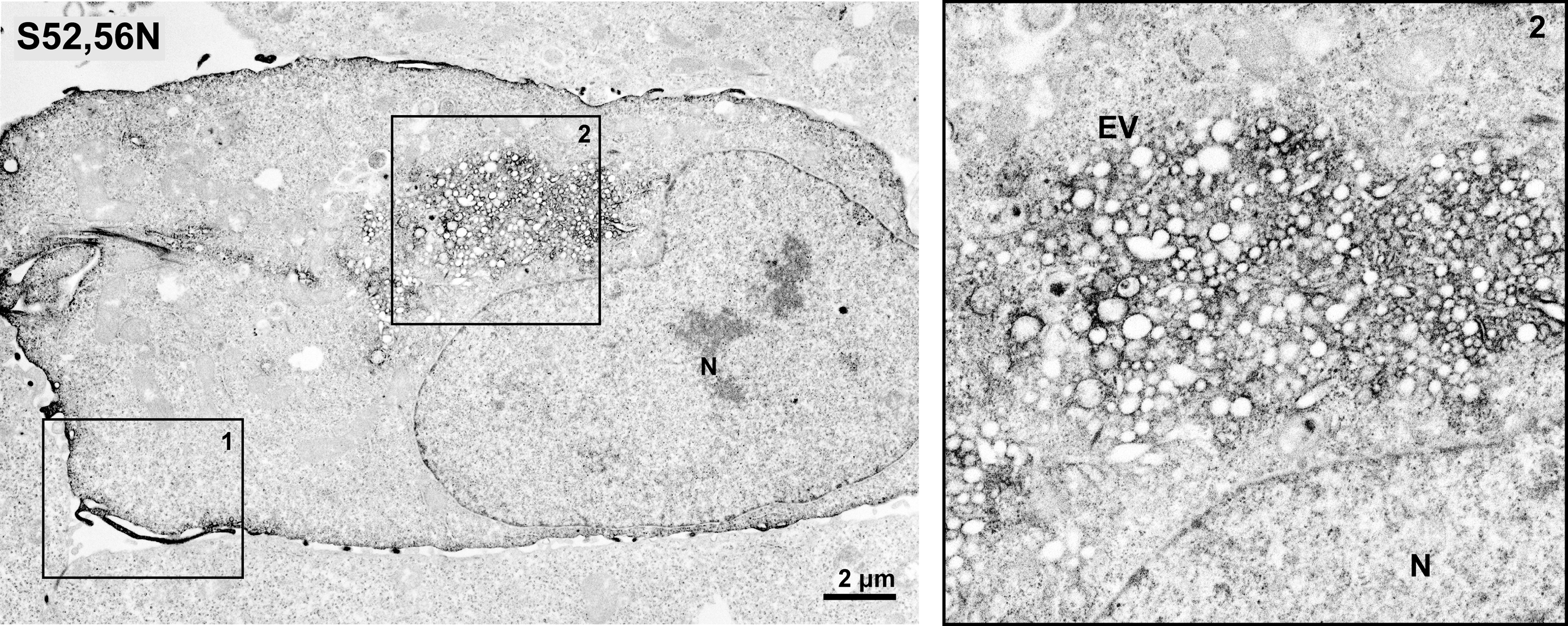

Supplement: S1 Fig — HeLa P4.R5 cells were transfected to express Vpu-S52,56N-APEX2. 24 hours later the cells were fixed before APEX2-dependent polymerization of DAB and osmium staining. Cells were embedded in resin and 70 nm sections collected and analysed by TEM. The mutant Vpu-S52,56N was localized to the plasma membrane region (region 1 is shown at higher resolution in Fig 3) but also induced formation of juxta-nuclear enlarged vesicles (EV, region 2), similar to the WT Vpu. (TIF) [file ppat.1009409.s001.tif]

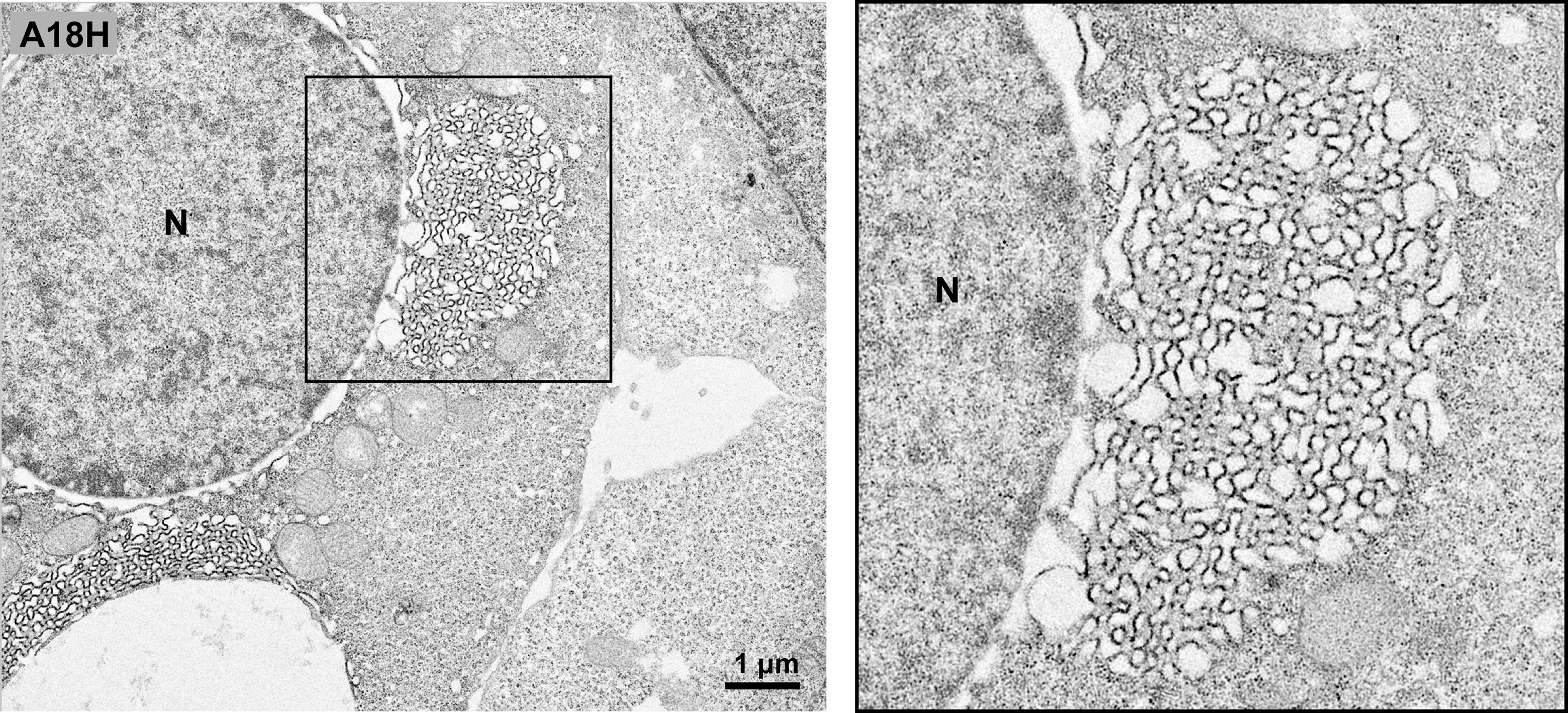

Supplement: S2 Fig — HeLa P4.R5 cells transfected to express VpHu-A18H construct bearing a C-terminal APEX2 tag were fixed before APEX2-dependent polymerization of DAB and osmium staining. Cells were embedded in resin and 70 nm sections collected and analysed by TEM. The endoplasmic reticulum-trapped mutant, A18H, was restricted to the nuclear envelope (NE) and ER and induced membrane reorganisation: when expressed at high levels, the nuclear envelope was distorted by the accumulation of convoluted, smooth membranes. (TIF) [file ppat.1009409.s002.tif]

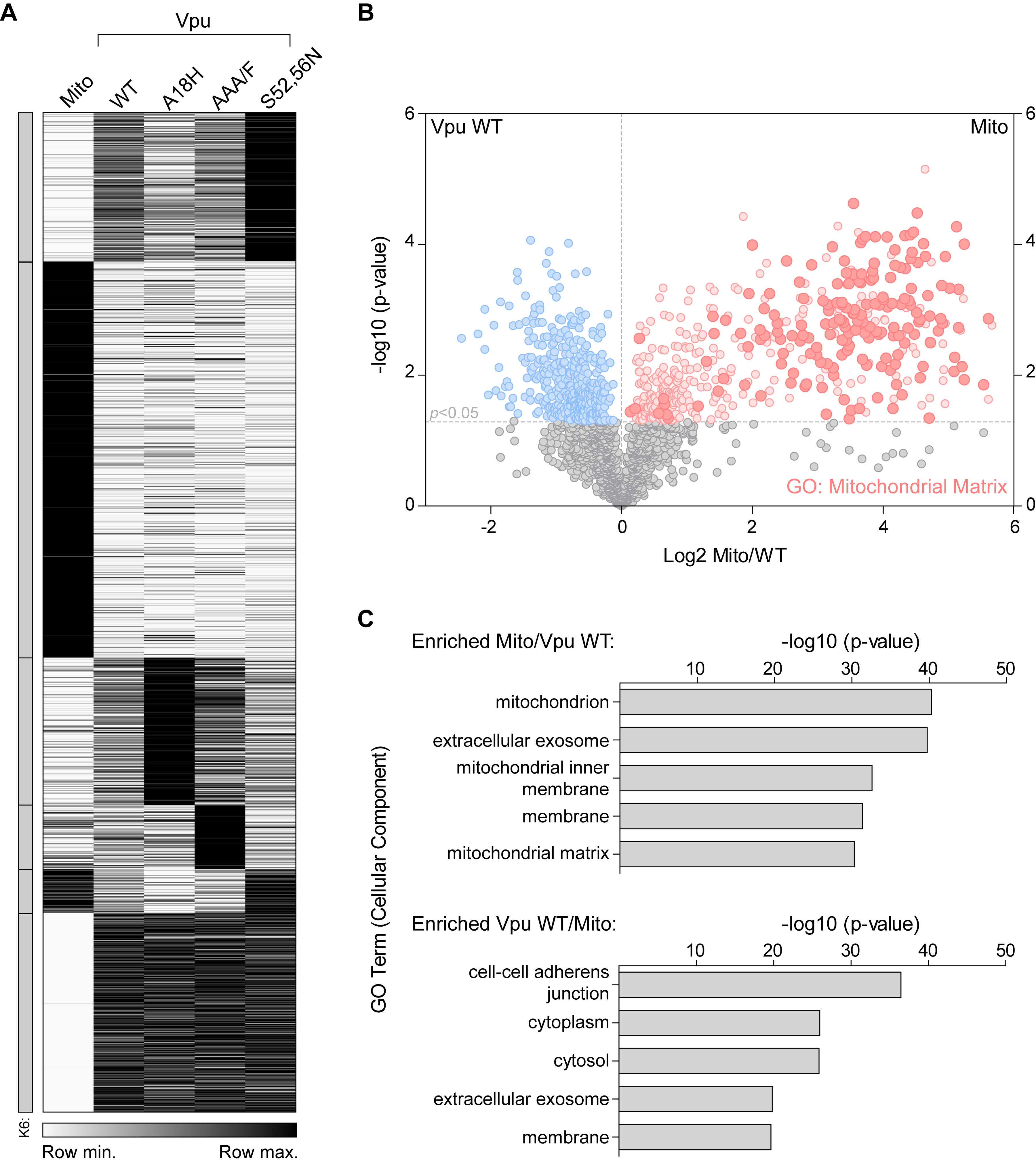

Supplement: S3 Fig — HeLa P4.R5 cells were transfected to express Mito (control) or Vpu constructs bearing C-terminal APEX2 tags, in duplicate. Following proximity biotinylation reactions, the biotinylated proteins were isolated and subject to quantitative mass spectrometry. (A) Heatmap showing relative protein abundance across Mito control and Vpu WT and mutant samples, sorted into 6 k-means clusters (cluster number derived from elbow method). (B) Volcano plot of proteins biotinylated by Vpu-APEX2 vs. Mito-APEX2 control. Mitochondrial proteins corresponding to GO term Mitochondrial Matrix are highlighted. The x-axis shows log2 fold change and y-axis -log10 p-value derived from Student’s t-test. (C) GO enrichment analysis of proteins significantly enriched by Mito-APEX compared to Vpu WT, and vice versa, the top 5 GO (cell component) terms are shown for each comparison. (TIF) [file ppat.1009409.s003.tif]

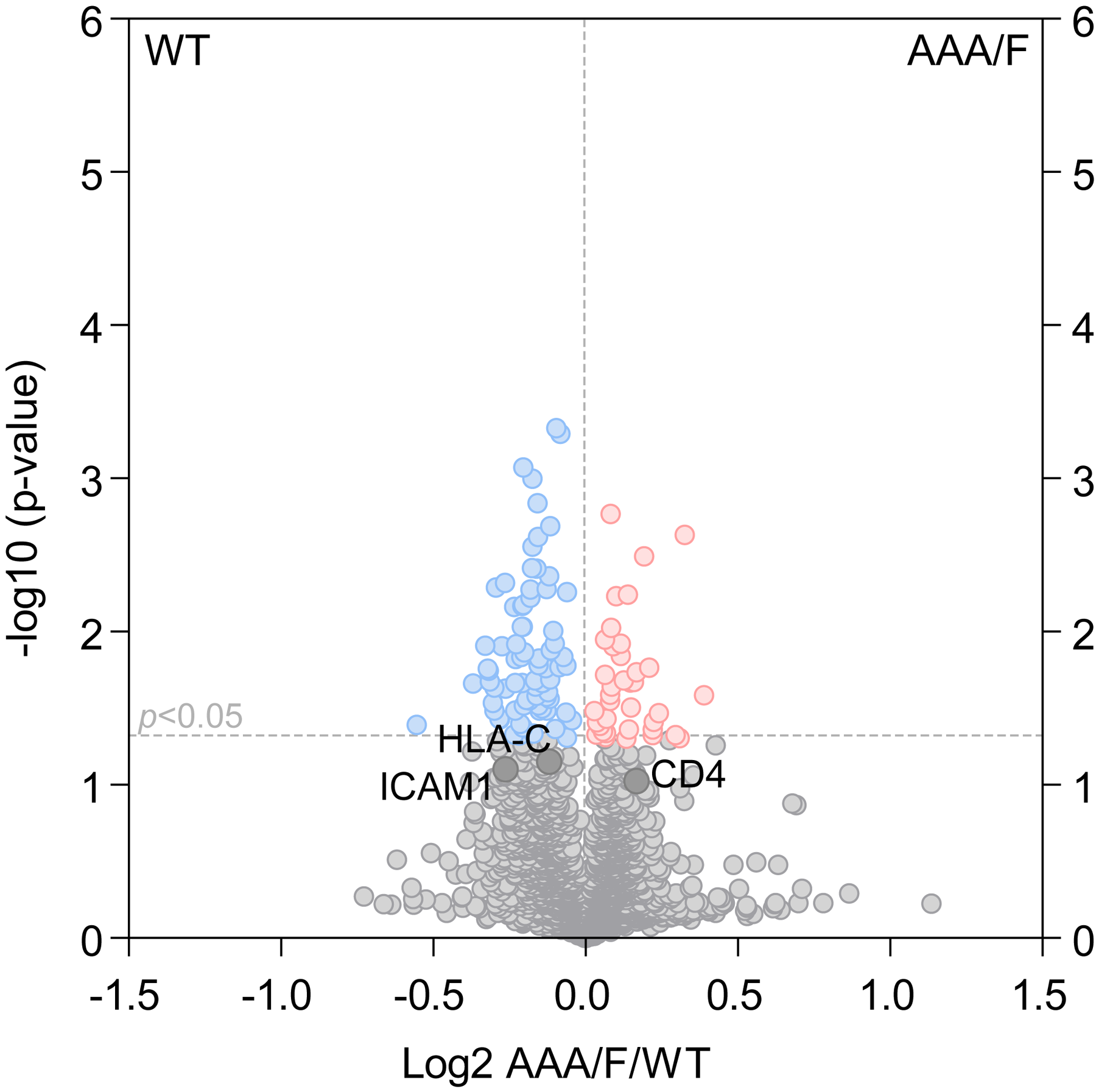

Supplement: S4 Fig — HeLa P4.R5 cells were transfected to express Vpu constructs bearing C-terminal APEX2 tags, in duplicate. Following proximity biotinylation reactions, the biotinylated proteins were isolated and subject to quantitative mass spectrometry. A volcano plot of protein enrichment in the presence of Vpu mutant AAA/F relative to the wild-type Vpu is shown, n = 2 experiments. Significantly enriched proteins highlighted in red and blue are derived from the Student’s t-test (p < 0.05). Known targets of Vpu are labelled. (TIF) [file ppat.1009409.s004.tif]

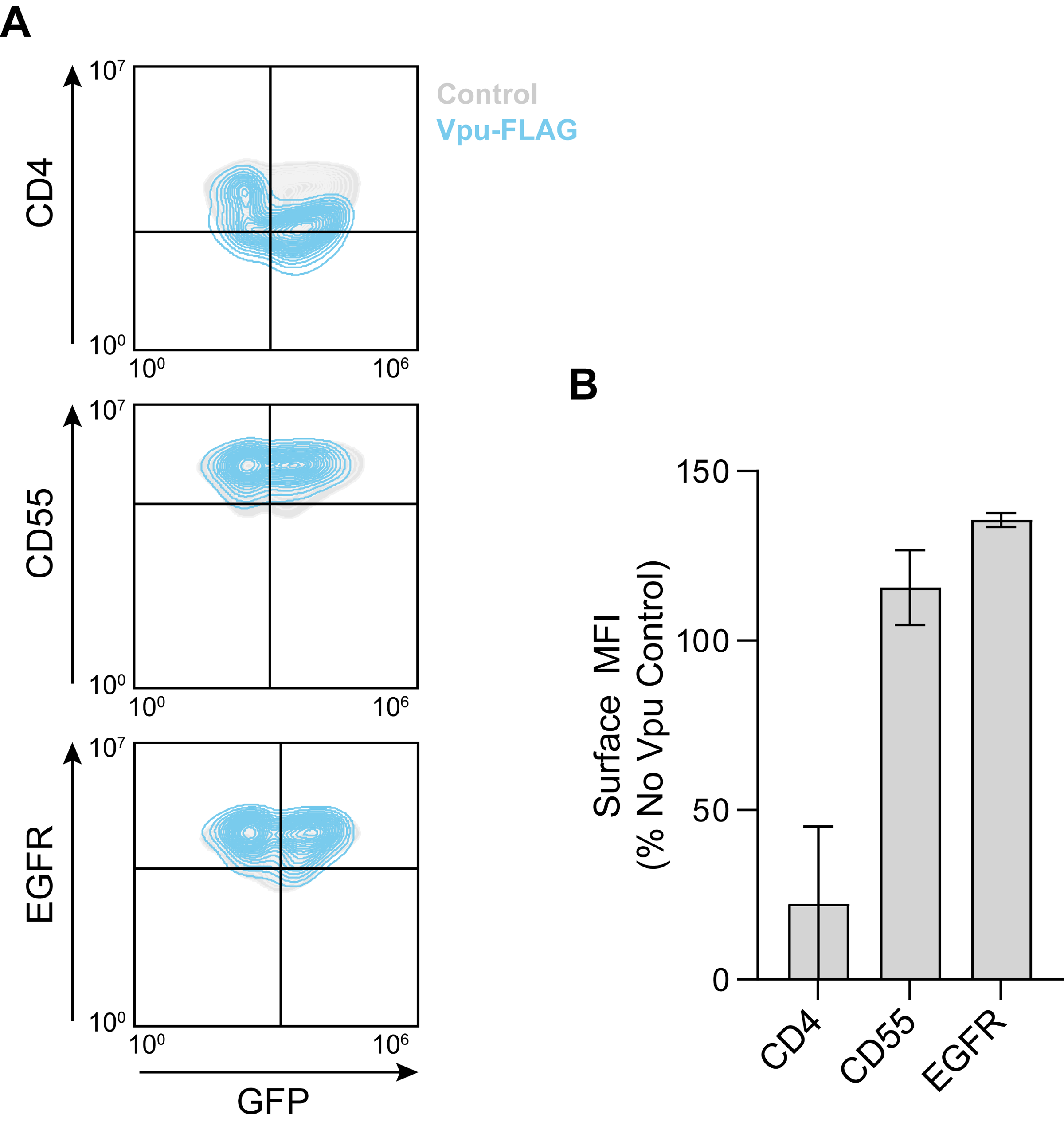

Supplement: S5 Fig — Cell-surface levels of CD55 and EGFR were measured in the presence of Vpu-FLAG by flow cytometry. Representative two-color flow plots are shown, CD4 was used as a positive control, GFP was used as a marker for transfection. Surface levels of CD4, CD55 and EGFR are expressed as % compared to control cells not expressing Vpu. Error bars represent standard deviation of n = 2 experiments. (TIF) [file ppat.1009409.s005.tif]

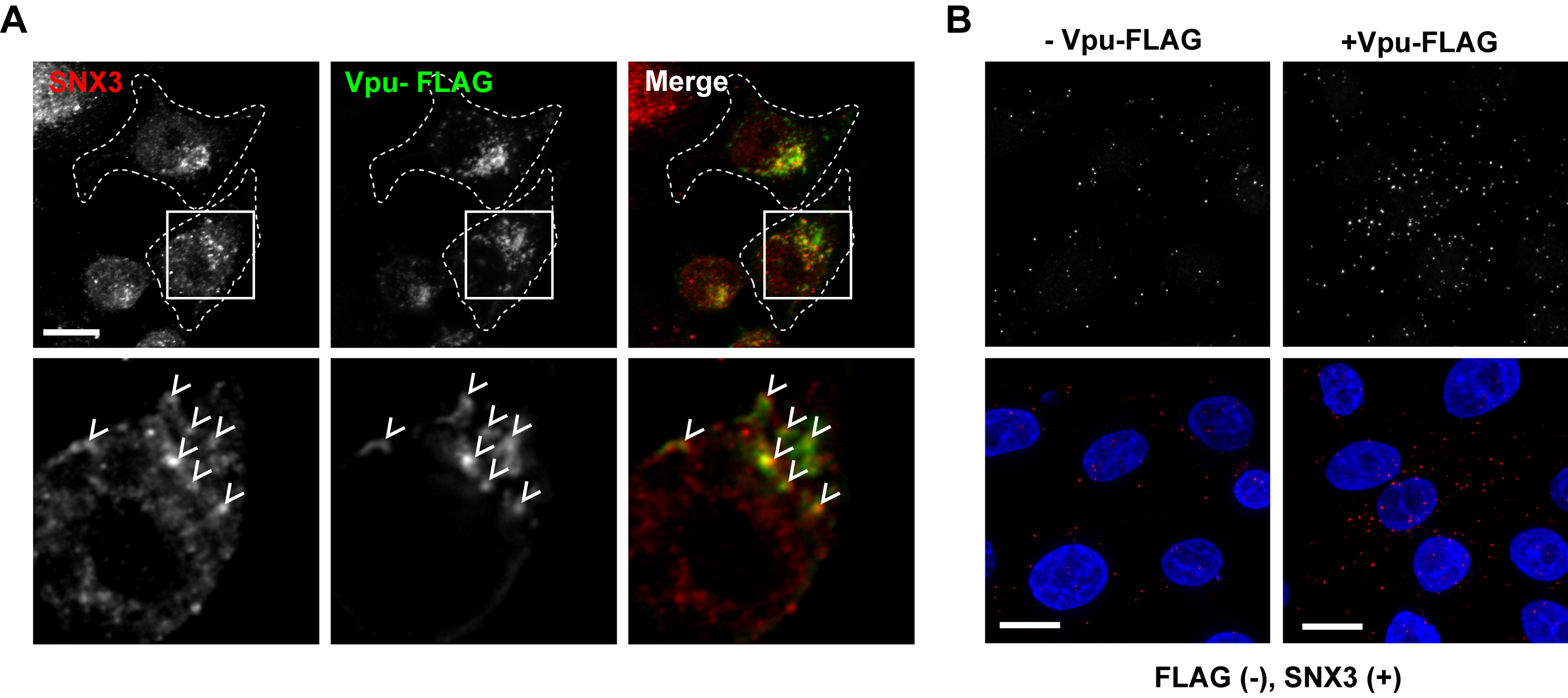

Supplement: S6 Fig — (A) HeLa P4.R5 cells transfected to express Vpu-FLAG were fixed and stained for endogenous SNX3 protein. Images are z-stack projections of full cell volumes; insets show single z-sections, with arrows indicating co-localized foci. Some punctate co-localization of Vpu and SNX3 was observed in the perinuclear region, in agreement with immunofluorescent stain of Vpu and retromer component Vps35. (B) PLA of Vpu:SNX3 proximity by fluorescence microscopy. HeLa P4.R5 cells transfected to express Vpu-FLAG or empty plasmid control were fixed and stained using anti-FLAG and SNX3 antibodies. The cells were stained with primer-conjugated secondary antibodies, followed by hybridization and PCR amplification with fluorescent probe. Red signal (over background) indicates close proximity (< 40 nm) between the proteins. Scale bars are 10 μm. (TIF) [file ppat.1009409.s006.tif]
